# A Layer 3→5 Circuit in Auditory Cortex That Contributes to Pre-pulse Inhibition of the Acoustic Startle Response

**DOI:** 10.3389/fncir.2020.553208

**Published:** 2020-10-30

**Authors:** Aldis P. Weible, Iryna Yavorska, Donna Kayal, Ulysses Duckler, Michael Wehr

**Affiliations:** Department of Psychology, Institute of Neuroscience, University of Oregon, Eugene, OR, United States

**Keywords:** auditory cortex, startle response modulation, canonical microcircuit, gap detection, layer 3

## Abstract

While connectivity within sensory cortical circuits has been studied extensively, how these connections contribute to perception and behavior is not well understood. Here we tested the role of a circuit between layers 3 and 5 of auditory cortex in sound detection. We measured sound detection using a common variant of pre-pulse inhibition of the acoustic startle response, in which a silent gap in background noise acts as a cue that attenuates startle. We used the Nr5a-Cre driver line, which we found drove expression in the auditory cortex restricted predominantly to layer 3. Photoactivation of these cells evoked short-latency, highly reliable spiking in downstream layer 5 neurons, and attenuated startle responses similarly to gaps in noise. Photosuppression of these cells did not affect behavioral gap detection. Our data provide the first demonstration that direct activation of auditory cortical neurons is sufficient to attenuate the acoustic startle response, similar to the detection of a sound.

## Introduction

How cortical circuits contribute to sensory perception and behavioral output remains a fundamental question in systems neuroscience. Building on more than a century of research into cortical physiology and connectivity (Grünbaum and Sherrington, 1902; Campbell, 1905; Ramon y Cajal, 1911), recent advances in the ability to manipulate and record from identified cell types have provided new insights into the computations performed by cortical circuits (for review, see Adesnik and Naka, 2018). Here, we focus on gap detection, a common variant of pre-pulse inhibition of the acoustic startle response, in which a silent gap inserted into continuous background noise acts as a cue that attenuates the startle reflex. Auditory cortex plays a critical role in reflex modification for gaps <32–64 ms in duration (Ison et al., 1991; Kelly et al., 1996; Bowen et al., 2003; Threlkeld et al., 2008; Masini et al., 2012; Weible et al., 2014b, 2020). Thus auditory cortical circuits contribute to the normal operation of this sensorimotor behavior. How sensory information flows through cortical circuits to mediate this behavior remains unknown.

A good starting point for understanding the flow of information through the cortex is the canonical cortical microcircuit, first proposed for the visual system (Gilbert and Wiesel, 1983; Douglas and Martin, 1991). In this model, sensory information from the thalamus first enters the cortex in layer 4, then ascends to superficial layers 2/3, then descends to deep layers 5/6, from where it either exits the cortex or closes the loop *via* an L6→L4 projection. One approach to understanding how information is transformed in the cortex is to measure neuronal responses at each stage of this circuit and test how manipulations at one stage impact responses at the next.

Since the original conception of the canonical cortical microcircuit, however, the picture has grown more complicated. For example, thalamocortical inputs are known to terminate throughout layers 1–6 (Harris and Shepherd, 2015). Numerous pathways that interconnect layers and sublayers have also been identified (Douglas and Martin, 2004; for review, see Adesnik and Naka, 2018). Furthermore, neighboring neurons within a given layer, even when focusing specifically on excitatory neurons and ignoring the broad diversity of interneurons, can exhibit different morphology, physiology, and connectivity patterns, and thus appear to be functionally distinct. These factors indicate that the components of cortical circuits are not individual layers, but rather classes of cells, which are typically intermingled with other cell classes within and among layers (Harris and Shepherd, 2015; Williamson and Polley, 2019). This suggests that recording from and manipulating specific cell classes, such as genetically identified cell types, maybe a useful strategy for understanding how cortical circuitry contributes to sensorimotor computation.

Here we tested the role of a genetically-identified class of cells in the mouse auditory cortex in brief gap detection. We used the Nr5a-Cre driver line (Harris et al., 2014, 2019; Tomorsky et al., 2017), which has not previously been characterized in the auditory cortex. Nr5a expression was restricted almost exclusively to layer 3. Photoactivation of these cells drove robust spiking in layer 5 cells, and also produced a behavioral inhibition of the startle response that was indistinguishable from that evoked by acoustic gaps in noise. A subset of layer 5 cells are known to send a major corticofugal projection to the inferior colliculus (IC), which is a critical component of the pre-pulse inhibition pathway (Li et al., 1998). This suggests a scenario in which Nr5a+ cells in layer 3 contribute to behavioral inhibition of the startle response by acting through layer 5 cortico-collicular cells in an L3→L5→IC circuit.

## Materials and Methods

### Mice

All procedures were performed in strict accordance with the National Institutes of Health guidelines, as approved by the University of Oregon Animal Care and Use Committee. We used +/+ offspring of crosses between hemizygous Tg(Nr5a1-Cre)2Lowl (“Nr5a”; 006364; The Jackson Laboratory, Bar Harbor, ME, USA) and homozygous CAG-ChR2-eYFP (“ChR2”; 012569, Ai32, The Jackson Laboratory, Bar Harbor, ME, USA), CAG-Arch-eGFP (“Arch”; 012735, Ai35D, The Jackson Laboratory, Bar Harbor, ME, USA), or Rosa-CAG-LSL-tdTomato-WPRE (“tdTomato”; Ai14, 007914, The Jackson Laboratory, Bar Harbor, ME, USA) lines. In these offspring, Cre-dependent ChR2 (behavior: *n* = 6 mice; physiology: *n* = 3), Arch (behavior: *n* = 9; physiology: *n* = 6), or tdTomato (*n* = 5) was expressed in Nr5a-positive (Nr5a+) pyramidal neurons. The mice are on a C57BL/6J background. For behavioral and electrophysiological experiments, we used Nr5a-negative (Nr5a−) littermates as controls (behavior: *n* = 9; physiology: *n* = 5).

### Anatomy

**Figure 1:**
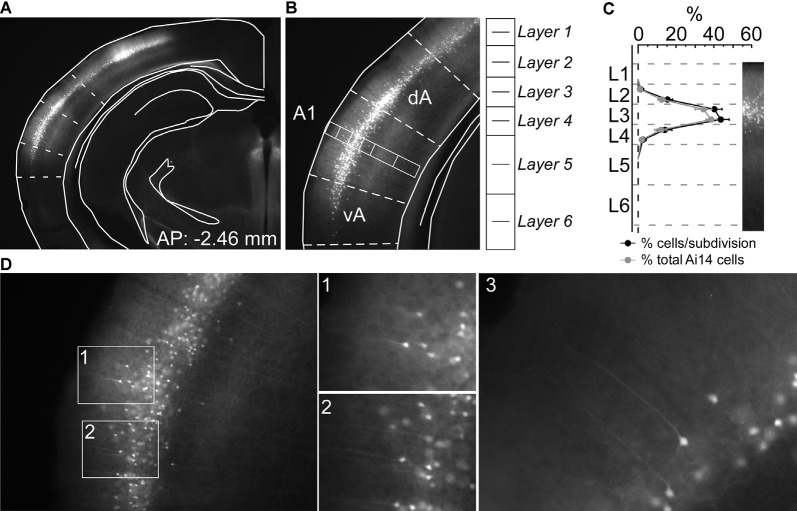
Nr5a-positive (Nr5a+) labeling is restricted primarily to layer 3. **(A)** tdTomato-labeled Nr5a+ cells in auditory cortex. We determined boundaries for the primary auditory cortex (A1) by aligning photomicrographs to atlas sections (Paxinos and Franklin, 2004). **(B)** We counted cells in a rectangular region spanning the depth of the cortex in the middle of A1. **(C)** We counted tdTomato-labeled Nr5a+ cells and CaMKII+ cells labeled by *in situ* hybridization from the rectangular sample regions. Penetrance (shown in black) is the proportion of tdTomato-labeled cells relative to CaMKII+ cells within each sublayer. The distribution of tdTomato-labeled cells across layers (shown in gray) is the percent of tdTomato-labeled cells in each sublayer relative to the total number of tdTomato-labeled cells, and sums to 100%. We did not quantify co-localization due to the quenching of the tdTomato signal and distortion of the tissue following *in situ* hybridization. Values are mean ± SEM. **(D)** Higher magnification views of labeled cells. Panels 1 and 2 are views of the insets in panel **(D)**. Panels 1–3 reveal pyramidal-shaped soma and apical dendrites extending toward layer 1. AP, anterior-posterior coordinate relative to Bregma; dA and vA, dorsal and ventral auditory cortex, respectively.

We crossed Nr5a-Cre mice with Cre-dependent tdTomato mice. We collected 50 μm coronal sections spanning auditory cortex from 5 Nr5a-tdTomato mice (8–12 weeks of age) and took photomicrographs of alternating sections on a Zeiss microscope using Zen software (Carl Zeiss Microscopy GmbH 2011). Seven of these sections were matched to the closest representative atlas section (Figure 1A; Paxinos and Franklin, 2004). We selected a rectangular region oriented perpendicular to the cortical surface, with a height extending from the pia to the external capsule, and a width 1/8th of this height, through the middle of primary auditory cortex (A1). To establish laminar boundaries as shown in Figure 1B, we subdivided this rectangular region as described by Anderson et al. (2009): layers 1, 2, 3, and 4 each represent 12.5% of the cortical thickness, and layers 5 and 6 each represent another 25%. We further subdivided each layer into two equal sublayers, to obtain finer-grained measures of penetrance with depth (Figure 1C). A sample count of cells was taken from the rectangular region. Counts of tdTomato-labeled cells were taken from 7 coronal sections, at 100 μm spacing. Cells were counted manually by two scorers and averaged. For three of the mice, we then performed *in situ* hybridization on the sections to label putative pyramidal neurons positive for Ca+/calmodulin protein kinase II (CaMKII). We used a digoxygenin (DIG)-labeled riboprobe (1:500), visualized by Anti Fluor-POD (1:1,000; Invitrogen, Cat. A21253) and Fluorescein (1:50; PerkinElmer, Cat. NEL741), as described previously (Weible et al., 2014a). We were not able to test for co-localization of tdTomato and CaMKII at cellular resolution, because *in situ* hybridization processing quenched the fluorescent tdTomato signal and also slightly distorted the tissue, which prevented precise spatial registration of before-and-after images. We therefore quantified CaMKII-labeled cells across lamina, using the same rectangular regions, to measure the penetrance of Nr5a+ cells proportional to the broader population of excitatory cells.

### Fiber Implantation

We administered atropine (0.03 mg/kg) pre-surgically to reduce respiratory irregularities. Mice were anesthetized with isoflurane (1.25–2.0%). One craniotomy was drilled in each hemisphere dorsal to auditory cortex (AP: −2.9 mm, ML: 4.4 mm, relative to Bregma) for the placement of 200 μm-diameter optic fibers (on the pial surface). We used cyanoacrylate and dental cement to secure the fibers to the skull. We administered ketoprofen (4.0 mg/kg) postoperatively. Mice were housed individually following the surgery and were given 7 days to recover.

### Behavioral Data Acquisition and Stimulus Delivery

All behavioral data were collected in a sound-attenuating chamber. Sounds were delivered from a free-field speaker directly facing the animal. The speaker was calibrated to within ±1 dB using a Brüel and Kjær 4939 microphone positioned where the ear would be but without the animal present. Mice were loosely restrained in a plastic tube (35 mm inner diameter, 1.5 mm wall thickness) affixed to a flat base. The tube was perforated (~3 mm diameter holes) to allow effective transmission of sound, with no more than 5 dB attenuation. The head was loosely clamped in position. An open slot along the top enabled access to the implanted fibers. Startle responses were measured with a piezo transducer positioned beneath the tube.

We inserted silent gaps into continuous 80 dB background white noise and measured how these gaps attenuated startle responses elicited by a 100 dB, 25 ms white noise burst. Gaps were 1, 2, 4, 8, 16, and 32 ms in duration, and there was a 50 ms interval between the end of the gap and the start of the startle stimulus. We also presented startle stimuli in isolation, without a gap (“gap-free” trials) to provide a baseline startle response. Each combination of gap duration and laser condition (see below) was presented 20 times per session, randomly interleaved and separated by a random inter-trial interval of 15 ± 5 s.

We separately examined how photostimulation and photosuppression of Nr5a+ cells affected behavior. For photostimulation, we used mice expressing Channelrhodopsin-2 (Nr5a-ChR2) and 445 nm wavelength laser diodes set to an output power of 50, 100, 200, or 300 mW/mm^2^. For photosuppression, we used mice expressing Archaerhodopsin (Nr5a-Arch) and 520 nm wavelength laser diodes with an output power of 300 mW/mm^2^. We chose these intensities based on the previous characterization of their spatial spread in the auditory cortex (Weible et al., 2014a,b). Laser-on trials were randomly interleaved with laser-off trials. For Nr5a-Arch mice, we delivered a 50 ms light pulse, starting 50 ms before the onset of the startle stimulus (see inset to Figure 4A). For Nr5a-ChR2 mice, we delivered a 25 ms pulse starting 50 ms before the startle on gap-free trials only (see inset to Figure 3A). To visually mask light delivery, we used strobe lights equipped with blue or green filters that pulsed continuously for the duration of the session. We confirmed the presence or absence of transgene expression (based on eGFP or eYFP fluorescence) and fiber placement in auditory cortex histologically after the experiment.

**Figure 2:**
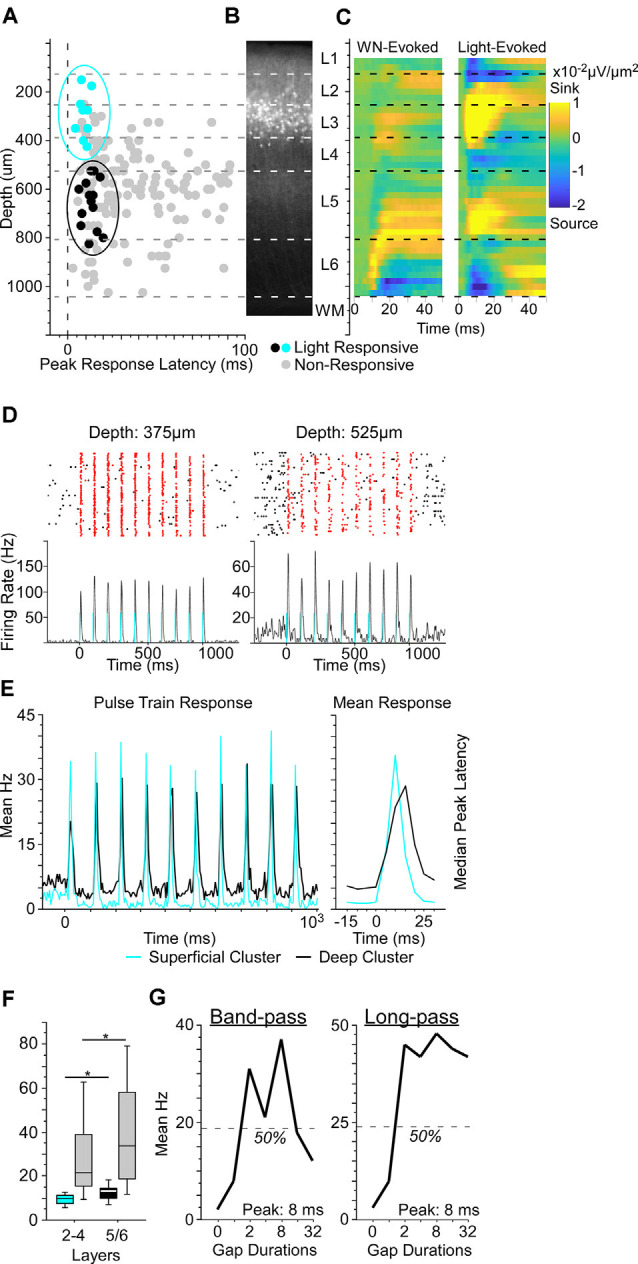
Optogenetically-identified Nr5a+ neurons in layer 3 respond to brief gaps in noise. **(A)** We established depths for 156 cells from three Nr5a-ChR2 mice. Of these, 23 were putative directly-activated cells (blue and black dots). For response criteria, see “Materials and Methods: Electrophysiology” section. Gray dots show the other 133 “non-responsive” cells; light intensity: 200 mW/mm^2^. When plotting depth by peak response latency, these 23 cells fell into one of two clusters, a superficial cluster (blue dots in the blue oval) with a median depth of 275 μm and a deep cluster (black dots in the black oval) with a median depth of 638 μm. **(B)** Cells in the superficial cluster matched the locations of tdTomato labeled cells from the Nr5a-Ai14 cross. **(C)** Left: white-noise-evoked current source density profile used for depth calibration. Right: both superficial and deep clusters corresponded to light-evoked current source density sinks (yellow hot-spots). Both panels are averages of 11 penetrations. **(D)** Two examples of neuronal responses to laser pulse-trains. Blue bars indicate light pulses (5 ms duration, 10 Hz). Spikes within 50 ms of light onset are shown in red. **(E)** The population-averaged responses from superficial and deep cluster cells were similar in shape but shifted in time, as illustrated pulse-by-pulse across the train (left) and in the mean pulse response (right). Color code as in panel **(A)**. **(F)** The median peak latency of cells in the superficial cluster preceded those of deeper responses by 3.5 ms. Non-responsive cells (gray bars) also showed a shift to longer median peak latency with depth (*p* = 0.03, *η*^2^ = 0.03). Boxes show median and interquartile range (IQR), whiskers show 10^th^ and 90^th^ percentiles. **(G)** Two examples of gap duration tuning curves from Nr5a+ cells (left, a band-pass tuned cell; right, a long-pass tuned cell). Nr5a+ cells were responsive to brief gaps and exhibited tuning similar to that of the broader population at the same depth. *p < 0.05.

**Figure 3:**
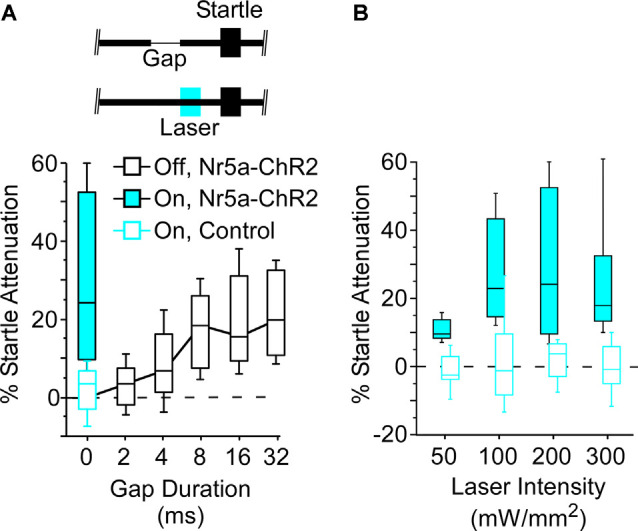
Optogenetic stimulation of Nr5a+ cells robustly attenuates the startle reflex. **(A)** Open black-box plot shows gap detection; longer gaps evoke progressively stronger startle attenuation. Boxes show median and IQR, whiskers show 10^th^ and 90^th^ percentiles. A 25 ms pulse of 445 nm blue light (200 mW/mm^2^), presented 50 ms before a 100 dB startle stimulus embedded in 80 dB continuous white noise without a gap, robustly attenuated evoked startle responses in Nr5a-ChR2 mice (filled blue box). No effect of light was seen with Nr5a- controls (open blue box). Inset depicts the laser pulse, background noise without a gap, and the startle stimulus. **(B)** Activation of Nr5a+ cells attenuated the startle response across a range of laser intensities (Wilcoxon signed-rank; 50 mW/mm^2^: *p* = 0.005; 100 mW/mm^2^: *p* = 0.003; 200 mW/mm^2^: *p* = 0.003; 300 mW/mm^2^: *p* = 0.008).

**Figure 4:**
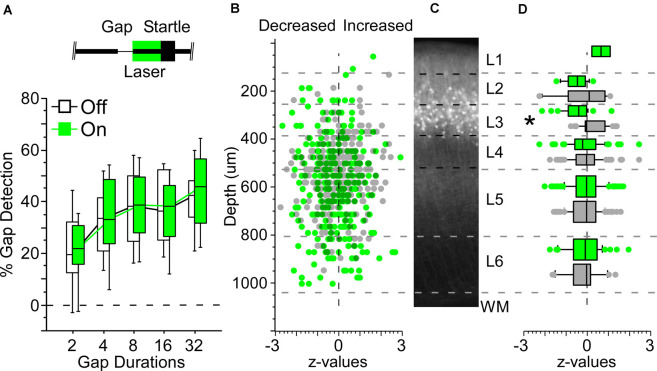
Illumination in the Nr5a-Arch line produced only weak suppression of spiking activity. **(A)** Box plots show gap detection, measured as percent startle attenuation; longer gaps evoked progressively stronger startle attenuation. Boxes show median and IQR, whiskers show 10^th^ and 90^th^ percentiles. A 50 ms pulse of 520 nm green light during the post-gap interval did not alter startle responses in Nr5a-Arch mice (green boxes; light intensity: 300 mW/mm^2^). Black boxes show gap detection on interleaved laser-off trials in the same mice. Inset depicts the laser pulse, background noise with a gap, and the startle stimulus. **(B)** Effect of illumination on each cell. Values are *z*-scores of firing rates averaged across all gap durations (laser-on relative to laser-off). Positive *z*-values indicate increased firing during illumination, negative *z*-values indicate suppression during illumination. Green dots are 267 cells from six Nr5a-Arch mice, gray dots are 287 cells from 4 Nr5a- control mice. **(C)** Expression pattern of tdTomato labeled cells from the Nr5a-Ai14 mouse for comparison. **(D)** Illumination significantly suppressed activity in Nr5a-Arch layer 3 neurons compared with those from controls. Same data as **(B)**, binned by layer. *p < 0.05.

### Behavioral Analysis

We quantified startle amplitudes by calculating the area of the rectified startle signal within a 100 ms window following onset of the startle stimulus, and taking the median across trials. Only sessions with significant gap detection for at least one gap duration (laser-off) were included for analysis. We tested whether gap detection was significant by comparing startle amplitudes associated with each gap duration to startle amplitudes on gap-free trials (paired *t*-test, *p* < 0.05). Because data for some gap durations were not normally distributed, group analyses were performed using non-parametric tests. For analysis of Nr5a-Arch data, laser-on and laser-off data within each session were normalized to that session’s median laser-off response. Comparisons between laser-on and laser-off conditions were performed with the Kruskal–Wallis test. For analysis of Nr5a-ChR2 data, we used the Wilcoxon signed-rank test to compare raw (non-normalized) laser-on and laser-off gap-free startles (because normalization of the data to the laser-off gap-free startles would artificially decrease variance). Data were collected from the same mouse for no more than four sessions, to minimize the likelihood of introducing experience-related shifts in startle behavior at brief gap durations (Swetter et al., 2010; Weible et al., 2014b).

### Electrophysiology

Mice were anesthetized with isoflurane (1.25–2.0%). A head post was secured to the skull and a mark was made on the skull over auditory cortex for a future craniotomy (AP: −2.9 mm, ML: 4.4 mm, relative to Bregma). Mice were housed individually following the surgery and were allowed at least 5 days of post-operative recovery. On the day of recording, mice were anesthetized with isoflurane (1.25–2.0%), the head was clamped with the head post, and a small craniotomy was made over auditory cortex (1 × 1 mm). Craniotomies were covered with a thin layer of agar and mice were allowed to recover for at least 1 h before recording.

All electrophysiological recordings were performed while the animal was awake and head-fixed on a styrofoam ball inside a double-walled acoustic isolation booth. Neurons in auditory cortex were recorded with a 32-channel silicon probe (25 μm spacing between sites, 750 μm shank, Neuronexus A1 × 32-Poly2–5mm-50s-177), Intan RHD2000 board, and Open Ephys software (Siegle et al., 2017). The silicon probe was positioned with a micromanipulator (MP-285, Sutter) orthogonal to the cortical surface such that the electrode sites spanned cortical layers. Spiking and local field potential signals were filtered online (600–6,000 and 0.1–400 Hz, respectively). Single neurons were identified offline using MClust spike sorting software (Redish, 2008) as described previously (Weible et al., 2020). To measure the depth of recorded cells, we used current-source density analysis of the local field potential evoked by 25 ms white noise bursts. We identified the robust sink with the shortest latency in upper L4 and assigned it a depth of 400 μm (Intskirveli and Metherate, 2012). We assigned the depths of individual neurons relative to this, based on the channel exhibiting the maximum waveform amplitude for each neuron. We assigned laminar boundaries using the same percentages as described above (see “Anatomy” section), applied to a total cortical thickness of 1,045 μm (Intskirveli and Metherate, 2012). This allowed us to relate recording depth to our histological analysis and laminar boundaries.

Neural data were collected during the presentation of gap-in-noise stimuli (Nr5a-Arch and Nr5a-ChR2), as well as light-pulse train stimuli for photo-identification of Nr5a+ neurons (Nr5a-ChR2 only). Laser intensities were 200 mW/mm^2^ for Nr5a-ChR2 recordings and 300 mW/mm^2^ for Nr5a-Arch recordings. The presentations of gaps-in-noise stimuli differed from the behavioral protocol in two respects. First, no startle stimuli were presented. Second, a shorter inter-trial interval was used (1 s vs. the 15 ± 5 s used during behavioral experiments). Recording sessions included 20 presentations each of gaps 1, 2, 4, 8, 16, and 32 ms in duration, as well as 20 gap-free trials. Gap Termination Responses (GTRs) were defined as a significant increase (paired *t*-test) in spiking activity during the 50 ms post-gap interval (i.e., following the resumption of noise) for at least two consecutive gap durations. Paired *t*-tests were also used to identify the within-cell effects of the laser. Between-group comparisons of spiking data were performed using non-parametric tests because some of the comparisons involved non-normally distributed data (Lilliefors), and because statistical power was comparable even when the underlying assumptions for the corresponding parametric analysis were met (Kitchen, 2009). GTRs were normalized using *z*-values (calculated relative to either the gap-free baseline interval or the laser-off trials of the same gap duration). We used the Kruskal–Wallis test (non-parametric alternative to the one-way ANOVA) to assess group differences across gap durations. We used the Wilcoxon rank-sum test (non-parametric alternative to the unpaired *t*-test) for laminar comparisons. We report effect sizes as eta-squared (*η*^2^; Lenhard and Lenhard, 2016). *η*^2^ varies between 0 and 1 and corresponds to the proportion of variance in the dependent variable explained by the independent variable. *η*^2^ values of 0.01–0.06 are generally considered to be small effects, *η*^2^ of 0.06–0.14 moderate effects, and *η*^2^ > 0.14 large effects.

We classified neurons with significant GTRs as all-pass, band-pass, short-pass, or long-pass based on their duration tuning curves (Casseday et al., 1994; Fuzessery and Hall, 1999). All-pass neurons responded above 50% of the peak firing rate across all gap durations. Band-pass neurons exhibited ≤50% of peak firing rate at durations both shorter and longer than the preferred duration. Short-pass neurons preferred brief durations and exhibited a decrease to ≤50% of the peak firing rate at longer durations. Long-pass neurons typically preferred longer durations and did not fire ≤50% of the peak firing rate at longer durations.

We used light-pulse trains to identify putative Nr5a+ neurons (Lima et al., 2009). Blue light pulses (445 nm, 5 ms duration, 200 mW/mm^2^) were delivered at a frequency of 10 Hz for 1 s. Twenty repetitions of this train were presented. To distinguish directly light-activated cells from downstream, indirectly-activated cells, we quantified light-evoked responses using three measures: response significance, peak response latency, and response reliability. We measured response significance as the *p*-value of a paired *t*-test comparing spiking activity in a 25 ms window following the onset of each light pulse to an equivalent laser-off baseline window. We measured response latency as the time-to-peak of the gaussian-smoothed (5 ms S.D.) trial-averaged firing rate following the onset of each light pulse. We measured response reliability as the proportion of trials on which light evoked 1 or more spikes in a 50 ms window following the onset of each light pulse. We could not identify any trends from the first to the last (tenth) pulse in the train that distinguished between putative directly-activated or indirectly-activated cells, so we averaged these measures across all pulses in the train. We also computed the Stimulus-Associated spike Latency Test (SALT) statistic (Kvitsiani et al., 2013), which was developed to detect light-evoked responses. We defined “putative directly-activated cells” as those that met one of two sets of criteria: (1) significance <0.0001, peak latency <20 ms, and reliability >0.5; or (2) significance <0.0001 and peak latency <15 ms. However, we note that putative directly-activated cells almost certainly included indirectly-activated cells in layer 5, as described below.

## Results

The detection of brief gaps in noise requires auditory cortex and specifically relies on spiking evoked by the end of the gap (termed the gap termination response, or GTR; Ison et al., 1991; Kelly et al., 1996; Bowen et al., 2003; Threlkeld et al., 2008; Weible et al., 2014b, 2020). The circuit mechanisms by which this activity contributes to gap detection remain unknown. Here we tested the role of a genetically-identified class of layer 3 pyramidal cells in this behavior. First, we characterized the expression pattern in the primary auditory cortex (A1). We then examined whether and how photoactivating (Nr5a-ChR2) and photosuppressing (Nr5a-Arch) these cells influenced startle responses and gap detection.

### Nr5a+ Cells Are Found in Layer 3

We first quantified the laminar expression pattern and penetrance in A1 for the Nr5a-Cre line. We counted tdTomato-labeled cells across layers in Nr5a-tdTomato mice (Figures 1A,B, *n*= 5 mice), and then counted CaMKII-positive (excitatory) cells in the same sections labeled by *in situ* hybridization (ISH). We did not measure whether individual cells showed co-localization of tdTomato and CaMKII (due to tissue processing effects; see “Materials and Methods” section), and therefore quantified penetrance as the percentage of tdTomato-labeled cells relative to the total number of excitatory neurons in each layer. The expression of the Nr5a-Cre line in A1 has not previously been described in detail. In primary visual cortex (V1), expression is restricted to layer 4 (Harris et al., 2014; Tomorsky et al., 2017). We found instead that expression in A1 was more superficial, with tdTomato labeled cell bodies limited predominantly to layer 3 (Figure 1C). Penetrance in layer 3 reached 42%. Immediately adjacent to layer 3, 15% of cells in sub-layer 2b and 17% of cells in sub-layer 4a expressed tdTomato (Figure 1C). Labeled cells were pyramidal-shaped with apical dendrites projecting toward the pial surface (see examples in Figure 1D), and with axonal projections descending to deep layers of cortex (Figures 1A,B,D). We only saw cells with pyramidal morphology, although we did not examine all labeled cells so we cannot rule out the existence of non-pyramidal Nr5a+ cells. No labeled neurons were observed in layers 1, 5, or 6.

### Layer 3 Nr5a+ Cells Strongly Drive Layer 5 Cells

What influence do these layer 3 Nr5a+ cells have on other cells in the cortical microcircuit during sensorimotor integration? To test this, we recorded from neurons across layers of auditory cortex with a linear silicon probe in Nr5a-ChR2 mice. To accurately measure the depth of recorded neurons, we used current-source density analysis to identify the sound-evoked robust short-latency sink in L4 (Intskirveli and Metherate, 2012). We recorded from a total of 192 cells (three mice, 14 penetrations), of which we could unambiguously assign precise laminar depth to 156 cells. Light evoked significant spiking responses in 48 of these cells, which were distributed throughout layers 2–6. The majority of these cells were activated with relatively long latency and low reliability, suggesting that they are likely to be indirectly-activated cells, postsynaptic to directly-activated cells expressing ChR2. Because Nr5a+ cells have a pyramidal morphology (Figure 1D), they are almost certainly excitatory and would be expected to broadly drive downstream neurons in the cortical circuit. In an attempt to distinguish between directly- and indirectly-activated neurons, we adapted a set of criteria based on the latency and reliability of light-evoked spiking responses (Lima et al., 2009). With these criteria, we identified 27 putative directly-activated cells from 3 Nr5a-ChR2 mice, of which we could unambiguously assign depths to 23 cells (Figure 2A; putative directly-activated cells: 27/192, or 14.1% of all cells recorded; 23/156, or 14.7% of cells with verifiable depths). These putative directly-activated cells were distributed across layers 2–5 (blue and black dots in Figure 2A) and formed two distinct spatial clusters: a superficial cluster (nine cells ranging in depth from 150 μm to 425 μm; 29% of cells in this depth range, blue dots in Figure 2A) and a deep cluster (14 cells ranging in depth from 525–825 μm; 13% of cells in this depth range, black dots in Figure 2A). The superficial cluster corresponded to the band of tdTomato-labeled cells centered in layer 3 (Figure 2B). Both the superficial and deep clusters corresponded to two robust light-evoked current-source density sinks in layers 3 and 5 (Figure 2C). However, the median latency to peak response of the deep cluster was significantly delayed relative to the superficial cluster (Figures 2D,E; latency difference was 3.5 ms, *p* = 0.04, *η*^2^ = 0.18, rank-sum test). Despite this delay at the group level, when cells were segregated by the depth we could not identify a set of spike latency or reliability criteria with which we could differentiate light-evoked responses from cells in the superficial and deep clusters. We also computed the SALT statistic, a latency-based test for detecting light-evoked responses (Kvitsiani et al., 2013). SALT values were significantly higher for putative directly-activated cells than for non-responsive cells (rank-sum *p* = 10^−5^), but cells in the superficial and deep clusters were not different from each other.

The proportion of superficial directly-activated cells mirrored the penetrance that we observed in Nr5a-tdTomato mice. Within layer 3, 33% of cells (5 of 15) were directly activated, compared with 42% that were labeled by tdTomato. Spanning layers 2b-4a, 23% of cells (7 of 31) were directly activated, compared with 29% labeled by tdTomato. Taken together, the correspondence with tdTomato-labeled cells in the superficial layers, the complete absence of tdTomato cells in the deeper layers, and the delayed response timing with depth provide strong evidence that the putative directly-activated cells in the superficial cluster (150–425 μm) were indeed directly-activated Nr5a-ChR2-expressing cells, whereas those in the deep cluster (525–825 μm) were downstream, indirectly-activated cells in layer 5. The short latency and high reliability of the light-evoked responses in these layer 5 cells suggest that they were driven by an exceptionally powerful monosynaptic projection from layer 3 Nr5a+ cells. This is consistent with the position of layer 5 immediately downstream of layer 3 in the canonical cortical circuit (Gilbert and Wiesel, 1983; Douglas and Martin, 1991, 2004). The fact that these two groups of cells were not separable based on the timing or reliability of light-evoked spikes (and could only be segregated by depth) provides an important and cautionary note about the limitations of these measures for accurately classifying optogenetically-tagged cells. For example, a recording technique with less spatial accuracy (such as chronically-implanted tetrodes) could potentially yield false-positive classification, at least with populations of strongly-connected excitatory neurons like those described here.

### Nr5a+ Cells Show Typical Responses to Gap Stimuli

Putative Nr5a+ cells and neighboring Nr5a- cells recorded in layers 2b-4a responded similarly to brief gaps in noise. The proportion of Nr5a+ cells exhibiting GTRs did not differ from neighboring cells (6/9 Nr5a+ cells vs. 15/39 Nr5a− cells; *χ*^2^ = 2.36, *p* = 0.12). Tuning properties of the observed GTRs also did not differ between Nr5a+ and neighboring Nr5a− cells. The proportion of band-pass and long-pass tuned responses was comparable between groups (Nr5a+: band-pass *n* = 2, long-pass *n* = 4; Nr5a−: band-pass *n* = 3, long-pass *n* = 12; *χ*^2^ = 0.42, *p* = 0.51; for examples, see Figure 2G), as was the median preferred gap duration (Nr5a+: 32 ms; Nr5a−: 20 ms; *p* = 0.13 rank-sum). No all-pass or short-pass responses were observed in either group.

### Nr5a+ Cells Drive Behavioral Startle Attenuation

Next, we asked whether the stimulation of these Nr5a+ cells would directly impact behavior. For this, we turned to a well-established sensorimotor paradigm: attenuation of the acoustic startle response. Auditory cortex makes a critical contribution to this behavior. A brief gap in continuous background noise attenuates the startle reflex, and the GTRs of auditory cortical neurons are required for this to occur (Weible et al., 2014b, 2020). The amount of attenuation increases with gap duration. We found that optogenetic stimulation of Nr5a+ cells preceding the startle pulse on gap-free trials strongly attenuated the startle reflex (Figure 3A, filled blue box; laser intensity: 200 mW/mm^2^). This attenuation was comparable to that seen following the longest gap duration presented (32 ms). Illumination had no effect in control mice that did not express ChR2 (Figure 3A, open blue box), ruling out artifactual attenuation due to visual or intracranial detection of the light pulses. Thus, stimulation of the Nr5a+ cells alone was sufficient to attenuate the startle reflex.

Startle attenuation was significant across a range of laser intensities (Figure 3B; range: 50–300 mW/mm^2^). Because a subset of layer 5 cells project corticofugally to the inferior colliculus (IC), a brain region known to be critically involved in startle attenuation, we speculate that activation of Nr5a+ neurons in layer 3, which in turn activates layer 5 cells (Figure 2), could act *via* an L3→L5→IC pathway.

### Suppression of Nr5a+ Cells Does Not Affect Gap Detection

Because Nr5a+ cells appeared to be sufficient for attenuating the startle response, we next asked whether optogenetic suppression of these cells could interfere with the normal startle attenuation evoked by gaps in background noise. We used Nr5a-Arch mice that expressed Archaerhodopsin in Nr5a+ cells and found that optogenetic suppression had no discernable impact on behavior (Figure 4A). Illumination in control mice also did not affect behavior (data not shown).

To test whether illumination caused optogenetic suppression of spiking in Nr5a+ cells, we recorded from neurons in all layers of auditory cortex with a linear silicon probe in Nr5a-Arch mice. We verified transgene expression by fluorescence in all mice histologically after the recordings. We recorded the activity of 350 neurons from six Nr5a-Arch mice and 340 neurons from four Nr5a- control mice while presenting gap-in-noise stimuli. Of these, we established reliable depths for 271 and 291 cells based on current-source density analysis, respectively (Figures 4B,C). Across all layers, illumination did not produce a significant change in activity in cells from Nr5a-Arch mice relative to controls, although there was a trend toward reduced activity (*p* = 0.08, *η*^2^ = 0.005, rank-sum). The same analysis performed layer-by-layer revealed a significant decrease in activity (*z*-scores) with illumination in cells from Nr5a-Arch mice in layer 3 relative to controls (Figure 4D; *p* = 0.0004, *η*^2^ = 0.22, rank-sum). This decrease in layer 3 activity was only significant for non-GTR cells, though a trend was also observed for GTR cells (*p* = 0.002, *η*^2^ = 0.2 and *p* = 0.053, respectively, rank-sum). We conclude that although Arch was indeed expressed in Nr5a+ cells and could modestly suppress their spiking activity, this optogenetic suppression was not robust enough to test whether gap responses in these neurons are necessary for startle attenuation.

## Discussion

Here, we identified a potent L3→L5 component of the canonical microcircuit in auditory cortex that contributes to behavioral startle attenuation. We found that Nr5a+ cells were pyramidal neurons located predominantly in layer 3 of auditory cortex, which showed gap responses similar in all major respects to those of other cortical neurons. Photoactivation of these layer 3 cells evoked short-latency and highly reliable spiking in layer 5 neurons, and robustly attenuated startle responses even at the lowest laser intensity tested. This behavioral inhibition of the startle response was indistinguishable from that evoked by acoustic gaps in noise. It therefore seems likely that some or all of these synaptically-driven layer 5 cells project corticofugally to inferior colliculus, which is a critical component of the pre-pulse inhibition pathway. These results are thus consistent with a scenario in which Nr5a+ cells in layer 3 contribute to behavioral inhibition of the startle response by acting through layer 5 cortico-collicular cells in an L3→L5→IC circuit.

Interestingly, Nr5a expression in mouse visual cortex is predominantly found in layer 4 (Bolser, 2004; Harris et al., 2014; Oh et al., 2014; Tomorsky et al., 2017), in contrast to our findings that these cells are primarily found in layer 3 of auditory cortex. The pial branching patterns of apical dendrites, axonal projections to deep layers, and strong synaptic activation of layer 5 neurons provide anatomical and physiological evidence consistent with the identification of Nr5a+ cells as layer 3 pyramidal neurons. Although layer 4 neurons in mouse sensory cortex do project to layer 5, the axonal projection from layer 2/3 to layer 5 is potent and densely branched, and is a prominent and consistent feature of cortical circuits across areas and species (Thomson and Bannister, 2003; Douglas and Martin, 2004; Harris and Shepherd, 2015). Indeed, the strongest projection from layer 3 is to layer 5, and this projection is a hallmark of the canonical microcircuit (Harris and Shepherd, 2015). We independently confirmed the location of these neurons in layer 3 of auditory cortex using anatomical (tdTomato expression) and physiological (ChR2-tagging and current-source density) characterization. We used anatomical laminar boundaries established in CBA mice (Anderson et al., 2009), which could in principle differ from those in our C57BL/6J mice, but agreed with our physiological characterization. We conclude that the laminar expression pattern of Nr5a differs between mouse auditory and visual cortex, with layer 3 expression in auditory cortex and layer 4 expression in visual cortex. The expression patterns of other laminar markers such as cytochrome oxidase and acetylcholinesterase also differ between mouse auditory and visual cortex (Anderson et al., 2009), as does the distribution of thalamocortical input (Linden and Schreiner, 2003). Why the Nr5a gene (a transcription factor in nuclear receptor subfamily five group A) shows different laminar expression patterns across cortical areas is unknown.

The canonical cortical microcircuit was first described in the visual system, in which its feedforward LGN→L4→L2/3 pathway has a natural interpretation in terms of the hierarchical representation of visual features, from circularly-symmetric to simple-cell to complex-cell receptive fields (Gilbert and Wiesel, 1983; Douglas and Martin, 1991). An alternative account views the canonical microcircuit in terms of predictive coding (Bastos et al., 2012; Adesnik and Naka, 2018; Keller and Mrsic-Flogel, 2018). In this view, ascending information (L4→L2/3) encodes prediction error, and prediction error neurons in layer 2/3 in turn provide feedforward connections that update internal representation neurons in layer 5. Central to the idea of predictive coding is the role of internal representation neurons, such as those in layer 5, which send a suppressive descending projection that cancels the activity of prediction error neurons at lower levels, presumably by engaging local inhibitory circuits. This cancellation corresponds to the "explaining away" of successfully predicted information. Pre-pulse inhibition of the startle response has a natural interpretation in this framework. A gap in continuous background noise, randomly timed so as to be unpredictable, generates a prediction error that is manifested as gap-evoked spiking in layer 2/3 neurons. This prediction error updates the internal representation in layer 5 neurons *via* the potent L3→L5 pathway that we have identified here. In turn, the corticofugal projection from layer 5 to the inferior and superior colliculus is relayed through the pedunculopontine tegmental nucleus, which produces long-lasting inhibition of premotor neurons in the caudal pontine reticular nucleus that mediates the startle response (Fendt et al., 2001). This long-lasting inhibition can be thought of as the cancellation of activity corresponding to a predicted event. In this framework, direct activation of layer 3 neurons with ChR2 generates a prediction error that drives the representation of an anticipated event in layer 5 neurons, canceling the corresponding startle response.

In contrast, a more conventional view of the functional significance of pre-pulse inhibition is that it serves to protect stimulus recognition (Graham, 1979; Fendt et al., 2001). The duration of pre-pulse inhibition (~150 ms) corresponds to a period of pre-attentive processing during which an unanticipated stimulus is recognized. A startle response during this period would evoke widespread behavioral and neural effects, which could interfere with stimulus recognition; pre-pulse inhibition might thus serve to protect sensory processing. This reflects a trade-off between the importance of recognizing an unexpected stimulus, and the importance of a startle response (such as a jump or an eyeblink) to avoid potential impact. Approaches that enable direct manipulation of the L3→L5 pathway, such as that described here or by others (Pluta et al., 2019), will help test the validity of these competing views.

Here we provide the first demonstration that direct activation of auditory cortical neurons is sufficient to attenuate the acoustic startle response. It remains unclear how an animal perceives the activation of layer 3 neurons (which in turn activates layer 5 and other downstream neurons). Because the ability of a pre-pulse stimulus (whether acoustic, tactile, or visual) to inhibit the startle response is closely tied to the extent to which the pre-pulse is consciously perceived (Fendt et al., 2001), pre-pulse inhibition has often been interpreted as an index of perception. For example, electrical stimulation of the cochlear nucleus can elicit pre-pulse inhibition, which led Zhang and Zhang (2010) to argue that electrical stimulation of the cochlear nucleus induces hearing. However, electrical stimulation of superior colliculus or the pedunculopontine tegmental nucleus can also elicit pre-pulse inhibition, and it seems less clear that stimulation of those multimodal structures would produce an acoustic percept. Nevertheless, animals can be trained to report electrical or optogenetic stimulation of remarkably small populations of neurons in the sensory cortex (Houweling and Brecht, 2008; Huber et al., 2008), and intracortical electrical stimulation of the auditory cortex in humans can evoke the auditory perception of sounds (Penfield and Perot, 1963; Fenoy et al., 2006). It, therefore, seems conceivable that optogenetic activation of layer 3 Nr5a+ neurons in the auditory cortex could evoke a phantom acoustic percept.

## Data Availability Statement

The raw data supporting the conclusions of this article will be made available by the authors, without undue reservation.

## Ethics Statement

The animal study was reviewed and approved by University of Oregon Institutional Animal Care and Use Committee.

## Author Contributions

AW, IY, DK, and UD performed research. AW and MW designed research, analyzed data, and wrote the article. All authors contributed to the article and approved the submitted version.

## Conflict of Interest

The authors declare that the research was conducted in the absence of any commercial or financial relationships that could be construed as a potential conflict of interest.
